# Determinants of maternal near miss among women in public hospital maternity wards in Northern Ethiopia: A facility based case-control study

**DOI:** 10.1371/journal.pone.0183886

**Published:** 2017-09-08

**Authors:** Dejene Ermias Mekango, Mussie Alemayehu, Gebremedhin Berhe Gebregergs, Araya Abrha Medhanyie, Gelila Goba

**Affiliations:** 1 Wachemo University, College of Medicine and Health Sciences, Department of Public Health, Hosanna, Ethiopia; 2 Mekelle University, College of Health Sciences, School of Public Health, Mekelle, Ethiopia; 3 University of Illinois at Chicago, Department of Obstetrics and Gynecology, Chicago, Illinois; National Academy of Medical Sciences, NEPAL

## Abstract

**Background:**

In Ethiopia, 20,000 women die each year from complications related to pregnancy, childbirth and post-partum. For every woman that dies, 20 more experience injury, infection, disease, or disability. “Maternal near miss” (MNM), defined by the World Health Organization (WHO) as *a woman who nearly dies*, *but survives a complication during pregnancy*, *childbirth or within 42 days of a termination*, is a proxy indicator of maternal mortality and quality of obstetric care. In Ethiopia, few studies have examined MNM. This study aims to identify determinants of MNM among a small population of women in Tigray, Ethiopia.

**Methods:**

Unmatched case-control study was conducted in hospitals in Tigray Region, Northern Ethiopia, from January 30-March 30, 2016. The sample included 103 cases and 205 controls recruited from among women seeking obstetric care at six (6) public hospitals. Clients with life-threatening obstetric complications, including hemorrhage, hypertensive diseases of pregnancy, dystocia, infection, and anemia or clinical signs of severe anemia (in women without hemorrhage) were taken as *cases* and those with normal obstetric outcomes were *controls*. Cases were selected based on proportion to size allocation while systematic sampling was employed for controls. Binary and multiple variable logistic regression (“odds ratio”) analyses were calculated at 95% CI.

**Results:**

Roughly 90% of cases and controls were married and 25% experienced their first pregnancy before the age of 16 years. About two-thirds of controls and 45.6% of cases had gestational ages between 37–41 weeks. Among cases, severe obstetric hemorrhage (44.7%), hypertensive disorders (38.8%), dystocia (17.5%), sepsis (9.7%) and severe anemia (2.9%) were leading causes of MNM. Histories of chronic maternal medical problems like hypertension, diabetes were reported in 55.3% of cases and 33.2% of controls. Women with no formal education [AOR = 3.2;95%CI:1.24, 8.12], being less than 16 years of age at first pregnancy [AOR = 2.5;95%CI:1.12,5.63], induced labor[AOR = 3.0; 95%CI:1.44, 6.17], history of cesarean section[AOR = 4.6; 95% CI: 1.98, 7.61] or chronic medical disorder[AOR = 3.5;95%CI:1.78, 6.93], and women who traveled more than 60 minutes before reaching their final place of care[AOR = 2.8;95% CI: 1.19,6.35] had higher odds of experiencing MNM.

**Conclusions:**

*Macro-developments* like increasing road and health facility access as well as expanding education will all help reduce MNM. Work should be continued to educate women and providers about common predictors of MNM like history of C-section and chronic illness as well as teenage pregnancy. These efforts should be carried out at the facility, community, and individual levels. Targeted follow-up with women with history of chronic disease and C-section could also help reduce MNM.

## Introduction

At the conclusion of the Millennium Development Goals (MDG) era, maternal mortality had declined by 45%, an impressive reduction, but well short of the 75% reduction targeted by the global community. Today, approximately 830 women die daily from pregnancy or childbirth globally. Almost all of these deaths occur in low-resource settings and most could be prevented[1]. Among developing regions, Sub-Saharan Africa (SSA) has the highest maternal mortality ratio (MMR) at 640 per 100,000 live births[1, 2]. Women in SSA have a 1 in 39 lifetime risk of dying in childbirth compared to 1 in 3,800 women in industrialized countries[3]. For every woman who dies of pregnancy complications, about 20 more–roughly 7 million women annually—experience injury, infection, disease, or disability[4].

With the global community seeking to reduce MMR to less than 70 per 100,000 live births by 2030 as part of the Sustainable Development Goals, much work remains[1].

The World Health Organization (WHO) defines *maternal near miss* (MNM) as *a woman who nearly dies*, *but survives a complication occurring during pregnancy*, *childbirth*, *or within 42 days of termination of pregnancy*[5]. MNM’s harmful consequences are numerous, including separating mothers and newborns, interfering with bonding, lengthy hospital stays and healthcare costs, and emotional distress. MNM is increasingly used as an indicator of the quality of obstetric care and clinical practice [2, 6, 7].

Ethiopia is one of five countries globally that account for half of maternal deaths [8]. About 20,000 women die each year in Ethiopia from complications during pregnancy, childbirth and in the post-partum period[9]. A study at Ayder Referral Hospital in Tigray, Ethiopia, revealed 204 severe acute maternal morbidities (SAMM) and 9 direct maternal deaths, equating to 22.7 SAMMs for every maternal death. The study also showed 101 SAMMs per 1,000 deliveries and an MMR of 427 per 100,000 live births[10].

Knowing the determinants of maternal mortality and morbidity is essential to designing effective interventions, [9], but reliable information is not available[11][12, 13]. Therefore, our study sheds light on causes of MNM, a proxy for maternal mortality, and can contribute to the design of interventions to minimize pregnancy-related complications and maternal death.

## Methods

The study was conducted in six (6) hospitals in Tigray, Ethiopia, from January 30 to March 30, 2016. Hospitals were randomly selected from 16 public hospitals in the region[14]. The study was a facility-based, unmatched case control design. Sample size was estimated using a double population proportion formula based on a study from Morocco that showed hypertensive disease contributing the most to MNM [15]. Based on the Morocco study, we hypothesized the proportion of chronic hypertension to be double in cases (63.9%) and controls (47%) at a 95% confidence level and 80% power of the test, with a 1:2 ratio for cases and controls. Final sample size was 308, of which 103 were cases and 205 controls.

We considered MNM as a condition meeting any of the five disease-specific criteria proposed by Filippi [16]. In sampled hospitals, using medical notes, any woman diagnosed with at least one of the following complications was considered as a case: severe obstetric hemorrhage leading to shock; hypertensive diseases of pregnancy, including eclampsia and severe preeclampsia; dystocia, including uterine rupture and impending rupture; infections, including hyper- or hypothermia or a clear source of infection and clinical signs of shock, and; anemia, including low hemoglobin (<6 g/dl) or clinical signs of severe anemia in women without hemorrhage. Women not meeting the above criteria were considered as controls. Cases were sequentially recruited whereas controls were selected through systematic sampling. Data was collected using a structured questionnaire, administered in-person by nurse midwives. Socio-demographic characteristics, obstetric history, and knowledge of pregnancy-related danger signs were collected.

Questionnaire was based on tools validated by the World Health Organization (WHO) and in different literature and adapted to include context-specific factors [11–13, 15, 17]. Questionnaire was prepared in English, translated to Tigrigna, and back-translated to English separately by two individuals to ensure consistency. Data was collected by 12 nurse midwives with experience in obstetric care. Data collection was supervised and data checked for consistency and completeness. Incomplete and unclear questionnaires were returned to interviewers to be completed.

### Data analysis

Data was entered, cleaned and analyzed using SPSS 20. Data was cleaned by running frequencies, cross-tabulation and sorting cases. Bar graphs and frequencies were used to represent results of categorical variables. Bivariate and multivariate logistic regression analyses were used to determine the association of independent variables with the dependent variable. Variables with p<0.25 in bivariate analysis were entered into a multivariate logistic regression model. Odds ratios with 95% confidence were computed to identify the presence and strength of associations, and statistical significance was declared if p<0.05 was found. The final model was checked using the Hosmer–Lemeshow goodness of fit test. Co-founders, interaction and multi-collinearity were checked to minimize bias.

### Ethics statement

Study protocol was approved by the Institutional Research Review Board of Mekelle University’s College of Health Sciences and Community Services Ethical Review Committee. Permission was obtained from Tigray Regional Health Bureau and participating hospitals. Informed verbal consent was obtained from participants prior to enrollment in the study. Participation in the study was voluntary and participants were informed of the right to withdraw from the study. Data collection was conducted confidentially and data de-identified, de-linked and stored in a secure location.

## Results

### Socio-demographic characteristics of study participants

A total of 308 participants were interviewed, with a response rate of 100%. The 20–29 age groups accounted for37.9% of cases and 31.7% of controls. Seventy-eight percent (78%) of controls and 57.3% of cases were from urban areas. The percentage of cases with no formal education was double that of controls. Nearly one-fourth of cases and 41% of controls had completed secondary education. Nearly double the percentage of cases had husbands who were farmers compared to controls (Table 1).

**Table 1:** Socio-demographic characteristics of mothers admitted to public hospitals, Tigray, Northern Ethiopia, 2016. (N = 308).

Variable	Category	Maternal Near Miss Status		
		Case n = 103(%)	Control n = 205(%)	Total N = 308(%)
**Age**				
	<20	20(19.4)	49(23.9)	69(22.4)
	20–29	39(37.9)	65(31.7)	104(33.8)
	30–39	11(10.7)	36(17.6)	47(15.3)
	40–49	33(32)	55(26.8)	88(28.6)
**Residence**				
	Rural	44(42.7)	45(22)	89(28.9)
	Urban	59(57.3)	160(78)	219(71.1)
**Maternal education**				
	No formal education	42(40.8)	36(17.6)	78(25.3)
	Primary	3(2.9)	13(6.3)	16(5.2)
	Secondary	32(31.1)	74(36.1)	106(34.4)
	More than secondary	24(23.3)	84(41)	108(35.1)
**Maternal occupation**				
	Farmer	28(27.2)	32(15.6)	60(19.5)
	Housewife	25(24.3)	77(37.6)	102(33.1)
	Government employee	21(20.4)	68(33.2)	89(28.9)
	Merchant	11(10.7)	9(4.4)	20(6.5)
	Unemployed	9(8.7)	11(5.4)	20(6.5)
	Student	9(8.7)	8(3.9)	17(5.5)
**Marital status**				
	Single	2(1.9)	7(3.4)	9(2.9)
	Married	92(89.3)	185(90.2)	277(89.9)
	Divorced	9(8.7)	12(5.9)	21(6.8)
	Widowed	0	1(0.5)	1(0.3)
**Husband education**				
	Illiterate	35(38.5)	71(36.8)	106(37.3)
	Literate	56(61.5)	122(63.2)	178(62.7)
**Husband occupation**				
	Farmer	39(41.9)	38(19.8)	77(27)
	Government employee	26(28)	79(41.1)	105(36.8)
	Merchant	23(24.7)	`65(33.9)	88(30.9)
	Unemployed	5(5.4)	1(0.5)	6(2.1)
**Monthly income**				
	<50 USD	35(34)	63(30.7)	98(31.8)
	50–100 USD	33(32)	67(32.7)	100(32.5)
	> = 150USD	35(34)	75(36.6)	110(35.7)

### Obstetric history

Approximately one-third (35.1%) of women were married before the age of 18 years and one-fourth had their first child before the age of 16 years. More than 40% of cases had five or more pregnancies compared to 20.5% of controls; roughly half of cases and controls had parity of 1–2. About two-thirds of controls and 45.6% of cases had gestational age between 37–41 weeks. Over three-fourths of controls and 52.4% of cases had no history of abortion, while 28.2% of cases and 17.1% of controls had one abortion. Among cases with history of abortion, 90% had a gestational age of 14–28 weeks; in contrast, 71% of controls had a gestational age of less than 14 weeks. Approximately, 80% of cases and controls had previously given birth at a facility. One-third (32.3%) of cases and 15.7% of controls had birth intervals of less than two years prior to the current delivery. Besides, nearly five-in-ten (44.7%) of the cases and two-in-ten (20%) of the controls had induction of labour (Table 2).

**Table 2:** Obstetric characteristics of mothers admitted in public hospitals, Tigray, Northern Ethiopia, 2016. (N = 308).

Variable	Category	Maternal Near Miss Status		
		Case n = 103 (%)	Control n = 205 (%)	Total N = 308(%)
**Age at 1^st^ marriage**				
	< = 18 year	40(38.8)	68(33.2)	108(35.1)
	19–24 year	36(35)	91(44.4)	127(41.2)
	> = 25 year	27(26.2)	46(22.4)	73(23.7)
**Age at 1^st^ pregnancy**				
	<16 year	40(38.8)	38(18.5)	78(25.3)
	16–19 year	31(30.1)	68(33.2)	99(32.1)
	> = 20 year	32(31.1)	99(48.3)	131(42.5)
**Gravidity**				
	1–2	40(38.8)	97(47.3)	137(44.5)
	3–4	20(19.4)	66(32.2)	86(27.9)
	> = 5	43(41.7)	42(20.5)	85(27.6)
**Parity**				
	0	4(3.9)	1(0.5)	5(1.6)
	1–2	45(43.7)	112(54.6)	157(51)
	3–4	23(22.3)	57(27.8)	80(26)
	> = 5	31(30.1)	35(17.1)	66(21.4)
GA* at delivery				
	<37 week	34(33)	54(26.3)	88(28.6)
	37–41 week	47(45.6)	123(60)	170(55.2)
	> = 42 week	22(21.4)	28(13.7)	50(16.2)
**Birth Interval prior to current pregnancy**				
	<2 year	30 (32.3)	22(15.7)	52(22.3)
	2 year	15(16.1)	73(52.1)	88(37.8)
	3 year	19(20.4)	30(21.4)	49(21)
	> = 4 year	29(31.2)	15(10.7)	44(18.9)
**Place of last birth**				
	Home	21(22.6)	29(20.7)	50(21.5)
	Health facility	72(77.4)	111(79.3)	183(78.5)
**Previous obstetric complication**				
	Yes	64(68.8)	79(56.4)	143(61.4)
	No	29(31.2)	61(43.6)	90(38.6)
**Induction of labor**				
	Yes	46(44.7)	41(20)	87(28.2)
	No	57(55.3)	164(80)	221(71.8)
**History of abortion**				
	0	54(52.4)	161(78.5)	215(69.8)
	1	29(28.2)	35(17.1)	64(20.8)
	> = 2	20(19.4)	9(4.4)	29(9.4)
**Gestational age at abortion**				
	<14 weeks	5(10.2)	31(70.5)	36(38.7)
	14–28 weeks	44(89.8)	13(29.5)	57(61.3)
**Neonatal condition at birth**				
	Live birth	82(79.6)	173(84.4)	225(82.8)
	Still birth	21(20.4)	32(15.6)	53(17.2)
**Birth weight**				
	<1.5 kg	22(24.4)	52(26.4)	74(25.8)
	1.5–2.49 kg	30(33.3)	76(38.6)	106(36.9)
	2.5–4 kg	25(27.8)	53(26.9)	78(27.2)
	>4 kg	13(14.4)	16(8.1)	29(10.1)

*GA (Gestational Age)

### Health related characteristics of respondents

History of chronic medical disorder, including hypertension, diabetes mellitus (DM) and cardiovascular disease (CVD) was reported in 55.3% of cases compared to 33.2% of controls. Nearly half (46.6%) of cases and 19.5% of controls had one previous C-section. Among women with history of C-section, 40.8% of cases had two or more C-sections compared to 19% of controls.

Only 7.8% of cases and 1% of controls had no ANC follow-up. Nearly half (48.1%) of cases started ANC between 24–28 weeks of gestation, considerably later than the 90% of controls started ANC before 16 weeks of age. Skilled birth attendants delivered 97.4% of all mothers and more than half (52.4%) of cases and 78.5% of controls delivered at public hospitals. Half of cases and one-third of controls were delivered by emergency C-section. There was not a great deal of variation in how cases and controls were transported to care, though 60% of cases experienced a delay of greater than 60 minutes in arriving at care compared to 40% of controls. One-third (32.5%) of cases were referred from health centers, 36.2% from health posts, and 30% self-referred. In contrast, 75% of controls were referred from a health center or private clinic (Table 3).

**Table 3:** Health-related characteristics of mothers admitted in public hospitals, Tigray, Northern Ethiopia, 2016. (N = 308).

Variable	Category	Maternal Near Miss Status		
		Case n = 103 (%)	Control n = 205(%)	Total N = 308
**Previous C-section**				
	0	48(46.6)	146(71.2)	194(63)
	1	48(46.6)	40(19.5)	88(28.6)
	> = 2	7(6.8)	19(9.3)	26(8.4)
**ANC visit**				
	0	8(7.8)	2(1)	10(3.2)
	1	11(10.7)	2(1)	13(4.2)
	2–3	54(52.4)	6(2.9)	60(19.5)
	> = 4	30(29.1)	195(95.1)	225(73.1)
First trimester Gestational Age				
	<16 week	15(19)	174(89.7)	198(69.2)
	16–20 week	19(24.1)	10(5.2)	29(10.6)
	24–28 week	38(48.1)	10(5.2)	48(17.6)
	> = 32	7(8.9)	0	7(2.6)
**ANC received facility**				
	Health post	26(27.4)	5(2.5)	31(10.4)
	Health center	46(48.4)	78(38.4)	124(41.6)
	Public hospital	22(23.2)	102(50.2)	124(41.6)
	Private clinic	1(1.1)	18(8.9)	19(6.4)
**Place of current delivery**				
	Home	2(1.9)	1(0.5)	3(1)
	Health post	1(1)	1(0.5)	2(0.6)
	Health center	46(44.7)	42(20.5)	88(28.6)
	Public hospital	54(52.4)	161(78.5)	215(69.8)
**Birth attendants**				
	Self	6(5.8)	2(1)	8(2.6)
	SBA*	97(94.2)	203(99)	300(97.4)
**Mode of current delivery**				
	SVD^	18(17.5)	67(32.7)	85(27.6)
	Emergency C/S^∞^	51(49.5)	64(31.2)	115(37.3)
	Instrumental^α^	18(17.5)	41(20)	59(19.2)
	Elective C/S^∞^	16(15.5)	33(16.1)	49(15.9)
**Type of current delivery**				
	Singleton	103(100)	203(99)	306(99.4)
	> = twins	0	2(1)	2(0.6)
**Delay at home before decide to seek obstetric care**				
	<24 hour	30(29.1)	99(48.3)	129(41.9)
	24–36 hour	38(36.9)	66(32.2)	104(33.8)
	>36 hour	35(34)	40(19.5)	75(24.4)
**Delay on the way to final place of care**				
	<30 minute	19(18.4)	62(30.2)	81(27.3)
	30–60 minute	22(21.4)	60(29.3)	82(26.6)
	>60 minute	62(60.2)	83(40.5)	145(47.1)
**Body mass index**				
	<18.5	19(18.4)	41(20)	60(19.5)
	18.5–24.9	25(24.3)	104(50.7)	129(41.9)
	25–29.9	44(42.7)	48(23.4)	92(29.9)
	> = 30	15(14.6)	12(5.9)	27(8.8)
**Means of transport**				
	Ambulance	20(19.4)	47(22.9)	67(21.8)
	Rented transport	40(38.8)	75(36.6)	115(37.3)
	Personal vehicle	20(19.4)	46(22.4)	66(21.4)
	On foot	23(22.3)	37(18)	60(19.5)
**Source of referral**				
	Self	24(30)	3(6.2)	27(21.1)
	Health post	29(36.2)	7(14.6)	36(28.1)
	Health center	26(32.5)	28(58.3)	54(42.2)
	Hospital	0	2(4.2)	2(1.6)
	Private clinic	1(1.2)	8(16.7)	9(7)

^SVD (Spontaneous Vaginal Delivery),

* SBA (Skilled Birth Attendants),

^∞^ C/S (Caesarian Section),

^α^ Instrumental (vacuum, forceps)

### Clinical characteristics of maternal near misses

As shown in Table 4, disease specific diagnostic criteria of MNM among cases included severe obstetric hemorrhage (44.7%), hypertensive disorders (38.8%), dystocia (17.5%), sepsis (9.7%) and severe anemia (2.9%). Of cases with hemorrhagic disorders, 32% were complicated by post-partum hemorrhage. Eleven (11) cases had more than one life-threatening obstetric complication (Table 4).

**Table 4:** Clinical characteristics of maternal near misses of mothers admitted in public hospitals, Tigray, Northern Ethiopia, 2016. (N = 308).

Maternal near miss events	MNM by condition (n = 103)
	n (%)
**Severe obstetric hemorrhage**	46(44.7)
Abruption placenta	8(7.8)
Placenta previa	5(4.9)
Retained placenta	14(13.6)
Uterine atony	13 (12.6)
Uterine inversion	4(3.9)
Disseminated Intra-vascular Coagulations	2(1.9)
**Hypertensive disorders**	40(38.8)
Severe pre-eclampsia	37(35.9)
Eclampsia	3(2.9)
Dystocia*	18 (17.5)
Prolonged/obstructed labor with previous C/S	12 (11.7)
Impeding uterine rupture	6(5.8)
**Infection or sepsis**	10(9.7)
**Severe anemia**	3(2.9)

Near misses may have multiple complications.

* difficulty in giving birth due to impending uterine rupture, prolonged/ obstructed labor with previous caesarian section

### Determinants of maternal near miss

Mothers who had no formal education had odds 3.2 times higher of experiencing MNM [AOR: 3.2, 95% CI: 1.24, 8.12]. Mothers who were less than 16 years of age at first pregnancy had odds 2.5 times higher [AOR = 2.5, 95% CI: 1.12, 5.63] of experiencing MNM and those for whom labor was induced had three times the odds [AOR = 3; 95% CI: 1.44, 6.17] of experiencing MNM. History of C-section was a strong determinant of MNM. Mothers with a prior C-section had odds four times higher [AOR = 4, 95% CI: 1.98, 7.61] of MNM and those with a history of chronic medical disorder had odds 3.5 times higher [AOR = 3.5, 95% CI: 1.78, 6.93]. Women that traveled more than 60 minutes to reach their final place of care had odds 2.8 times higher of experiencing MNM as those who traveled less than 30 minutes [AOR = 2.8, 95% CI:1.19,6.35] (Table 5).

**Table 5:** Determinants of maternal near miss among mothers admitted in public hospitals, Tigray, Northern Ethiopia, 2016. (N = 308).

Variables	Maternal near miss		COR[95%]	AOR[95%]
	Cases	Controls		
**Residence**				
Rural	44(42.7)	45(22.0)	2.65(1.59,4.42)*	1.4(0.58,3.45)
Urban	59(57.3)	160(78.0)	1	1
**Maternal education**				
No formal education	42(40.8)	36(17.6)	4.52(2.395,8.566)	**3.2(1.24,8.12)**^∞^
Primary	3(2.9)	13(6.3)	0.80(0.213,3.06)	0.6(0.09,3.90)
Secondary	32(31.1)	74(36.1)	1.5 (0.819,2.79)	1.47(0.63,3.41)
More than secondary	24(23.3)	84(41)	1	1
**Age at 1^st^ pregnancy**				
<16 year	40(38.8)	38(18.5)	3.25(1.79,5.91)*	**2.5(1.12,5.63)** ^∞^
16–19 year	31(30.1)	68(33.2)	1.41(0.78,2.52)*	1(0.48,2.32)
> = 20 year	32(31.1)	99(48.3)	1	1
**Gravida**				
1–2	40(38.8)	97(47.3)	1	1
3–4	20(19.4)	66(32.2)	0.7(0.39,1.36)	0.6(0.29,1.56)
> = 5	43(41.7)	42(20.5)	2.48(1.41,4.35)^	2.2(0.91,5.32)
**Induced labor**				
Yes	46(44.7)	41(20)	2.31(1.38,3.85)^	**3.0 (1.44,6.17)** ^∞^
No	57(55.3)	164(80)	1	1
History of chronic disorder^α^				
Yes	57(55.3)	68(33.2)	2.49(1.53,4)*	**3.5(1.78,6.93)** ^∞^
No	46(44.7)	137(66.8)	1	1
**History of C-section**				
0	48(46.6)	146(71.2)	1	1
> = 1	48(46.6)	40(19.5)	3.65(2.14, 6.21)	**4.0(1.98,7.61)** ^∞^
**Mode of current delivery**				
SVD^μ^	18(17.5)	67(32.7)	1	1
Emergency C-section	51(49.5)	64(31.2)	2.9(1.56,5.6)^	1.9(0.72,5.15)
Instrumental	18(17.5)	41(20)	1.63(0.764,3.49)	0.82(0.26,2.55)
Elective C-section	16(15.5)	33(16.1)	1.8(0.81,3.98)	1.1(0.36,3.56)
**History of multiple birth**				
Yes	55(58.5)	58(36.9)	2.4(1.42,4)^	1.6(0.86,3.22)
No	39(41.5)	99(63.1)	1	1
**Referral status**				
Yes	50(48.5)	54(26.3)	2(1.2,3.35)^	1.9(1,3.88)
No	53(51.5)	151(73.7)	1	1
**Delay at home before seeking obstetric care**				
<24 hour	30(29.1)	99(48.3)	1	1
24–36 hour	38(36.9)	66(32.2)	1.9(1.07,3.36) ^∞^	0.9(0.42,2.06)
>36 hour	35(34)	40(19.5)	2.88(1.5,5.31)^	1.8(0.77,4.17)
**Delay in reaching final place of care**				
<30 minute	19(18.4)	62(30.2)	1	1
30–60 minute	22(21.4)	60(29.3)	1.1(0.58,2.43)	0.5(0.19,1.37)
>60 minute	62(60.2)	83(40.5)	2.4(1.32,4.48) ^∞^	**2.8(1.19,6.35)** ^∞^

*Significant at p<0.05

^P < = 0.001,

^**∞**^P <0.0001

Chronic Medical Disorder^α^(Hypertension, Diabetes Mellitus, Cardiovascular Disease),

SVD^**μ**^(Spontaneous Vaginal Delivery)

## Discussion

In our study, lack of formal education, being less than 16 years of age atfirstpregnancy, induced labor, history of chronic medical conditions and C-section, and having to travel greater than 60 minutes to reach final place of care were all determinants of maternal near miss (MNM).

The observed association between lack of education and MNM is consistent with another study in Ethiopia[12], while a study from Brazil showed no significant association between education and MNM[18]. Education increases women’s access to relevant information and may facilitate the financial means required to pay for transportationto care. These factors could collectively influence mothers’ awareness of the need to seek better medical services, including delivering in health facilities.

Another predictor of MNM was being below 16 years of age at first pregnancy.Younger women are often not physically capable of childbearing. What’s more, pregnancy during teenage years frequently takes place outside the context of marriage, exposing women to adverse social consequences. The Government of Ethiopia is laudably targeting a reduction in teenage pregnancy from 12% to 3% by 2020, which will help[11,19,20,21].

Women with pre-existing chronic conditions had higher odds of MNM, which is consistent with studies in Brazil and the Netherlands[20, 22], though another study showed co-morbidities were not significantly associated with MNM[23]. For example, chronic hypertension considerably increases the risk of complications like superimposed pre-eclampsia, placental abruption, intra-uterine growth retardation and pre-term delivery [22, 24]. Chronic hypertension, DM and CVD may be indicators for referral to higher facilities. Encouraging screening for non-communicable diseaseswould be a good step to reducing MNM.

Our study showed the odds of MNM were four times higher among women with history of C-section, which is supported by studies in Brazil and the United States [25, 26], though a multicenter study showed C-section may be an acceptable tradeoff in cases of unfavorable cervical or fetal conditions [27].Interrupting pregnancy with a C-section increases the risk of infection, hemorrhage, thromboembolism and other complications, which can increase the chance of SAMM and MNM [23, 28]. Previous C-section may predispose mothers to placenta accrete in scar tissue as well as uterine rupture in attempted vaginal birth after C-section, both of which could lead to MNM.

Similar to other studies, our study showed induced labor was associated with MNM[25, 29]. Among women with induced labor, 33.3% progressed to uterine rupture.

In our study, women that were required to travel more than 60 minutes to their final point of care were at considerably higher odds of MNM, a finding consistent with studies in Nigeria and Morocco [15, 17]. Lack of available transport, particularly during night hours, travel distance and lack of roads, seeking care first ata facility ill-equipped to provide EMOC, and lack of recognition of the severity of complications, can all prolong care-seeking, leading toMNM [9, 11, 30–32].

## Conclusion

This study identified several factors correlated with women having maternal near miss (MNM). Lack ofeducation, having a first pregnancy before the age of 16 years, induced labor, history of chronic medical disorder as well as C-section, and delay in reaching final point of care were all predictors of MNM. Among these, history of chronic medical disorder and C-section, lack of education, and induced labor were the strongest determinants. The Government of Ethiopia must continue to address structural causes of MNM like lack of road and health facility access, lack of education, and teenage pregnancy. It must simultaneously help women and providers understand determinants of MNM at the facility, community, and individual levels. Targeted follow-up of women with history of chronic disease and C-section could be a practical way to reduce MNM by helping at-risk mothers plan for delivery. Subsidizing transportation to facilities could also be a key to reducing MNM. The data analyzed here are robust and provide information that can contribute to global maternal morbidity research agenda and guiding practice and policy about the most frequent complications and organ dysfunctions related to MNM.

## Strengths & limitations

Our study has several strengths, including employing a validated and standardized questionnaire that we tested and revised. Our study also used newly implemented Federal Ministry of Health (FMoH) guidelines on MNM to avoid misclassification. That said, the disease-specific criteria we used to classify MNM are not always straightforward. For instance, not all women with eclampsia experience MNM and not all women with an obstetric hemorrhage are critically ill, potentially resulting in over-reporting of MNM. Secondly, as an unmatched case-control design, our study may have included biases by not controlling for confounding by the matching factors.

## Supporting information

S1 File (SAV)
